# Inhibitory Effects of Sigma-2 Receptor Agonists on T Lymphocyte Activation

**DOI:** 10.3389/fphar.2013.00023

**Published:** 2013-03-13

**Authors:** Miguel A. Iñiguez, Carmen Punzón, Raquel Nieto, Javier Burgueño, José M. Vela, Manuel Fresno

**Affiliations:** ^1^Departamento de Biología Molecular and Instituto de Investigación Sanitaria Princesa, Centro de Biología Molecular Severo Ochoa (CSIC-UAM), Universidad Autónoma de MadridNicolás Cabrera, Madrid, Spain; ^2^Laboratorios EsteveBarcelona, Spain

**Keywords:** T lymphocytes, sigma receptor, transcriptional regulation, nuclear factor of activated T cells, nuclear factor-κB

## Abstract

Sigma (σ) receptor ligands are essentially known for their effects on the nervous system although recent studies have shown their potential effects modulating some other pathophysiological processes as cell proliferation, cancer, and the immune response. Here, we have analyzed the actions of σ-1 and σ-2 receptors ligands on T cell activation. Our results show that treatment of Jurkat T cells with σ-2 agonists decreased the induction of the expression of Interleukin (IL)-2, Tumor necrosis factor (TNF)-α, and Cyclooxygenase (COX)-2 by activated T cells in a dose-dependent manner. These effects take place at the transcriptional level since σ-2 agonists BD-737 and CB-184 diminished the activity of the promoters of those genes. Those immunosuppressive effects could be attributable to interference with transcription factor activation. Induced transcription mediated by Nuclear factor (NF)-κB or Nuclear Factor of Activated T cells (NFAT) was inhibited by σ-2 agonists. These effects seem to be specific for σ-2 agonists as no significant effects on T cell activation by σ-1 ligands PRE-084 and BD-1063 were found. Our results provide new insights into the immunomodulatory actions of σ ligands and describe a new property of σ-2 agonists, through inhibition of activation of transcription factors as NFAT by which these compounds are regulating gene expression. This may have important consequences on the possible therapeutic use of those compounds.

## Introduction

Sigma (σ) receptors were originally identified as members of the family of opioid receptors. Pharmacological studies have identified two subtypes of σ receptors, named σ-1 and σ-2 (reviewed in; Bowen, 2000; Su and Hayashi, 2003; Guitart et al., 2004). However, their endogenous ligands still remains unknown. Moreover, it has not been well defined which are the signal transduction pathways triggered by those receptors. Whereas the gene encoding the σ-1 receptor has been cloned and characterized (Hanner et al., 1996), the molecular nature of the σ-2 receptor is still not clearly defined. A recent report has identified the progesterone receptor membrane component 1 (PGRMC1) protein as the putative sigma-2 receptor (Xu et al., 2011). Sigma receptor ligands have been widely investigated for the treatment of brain related disorders (Bowen, 2000; Guitart et al., 2004) although recent studies have attributed to σ-1 and σ-2 ligands a therapeutic role in other pathologies as inflammatory diseases and cancer (Bourrie et al., 2004; Megalizzi et al., 2010; van Waarde et al., 2010).

These receptors are mainly expressed in a variety of distinct regions of the central nervous system but recent reports have also shown significant levels of sigma receptors in immune tissues. Thus, high-affinity σ receptors have been identified in human peripheral blood mononuclear cells (Su et al., 1988; Wolfe et al., 1988) and rat and mouse spleen (Carr et al., 1991; Liu et al., 1995) using selective radio-ligands. Although involvement of σ receptors in the regulation of inflammation and the immune response have been reported, few studies have addressed the selective effect of σ-1 and σ-2 ligands (either agonists or antagonists) on immune function so far. Most of the studies have been performed with dual σ-1 and σ-2 ligands, as SR31747, which binds with high-affinity to human and rat leukocytes and exhibit immunosuppressive properties *in vitro* and *in vivo*. This ligand suppresses mitogen-induced proliferation of mouse and human lymphocytes (Casellas et al., 1994) and stimulates LPS- or staphylococcal enterotoxin B-induced Interleukin (IL)-10 serum release, while concomitantly inhibiting Tumor necrosis factor (TNF)-α production “*in vivo*”(Bourrie et al., 1995, 1996; Derocq et al., 1995). “*In vitro*” studies have shown that SR31747A induces IL-10 and reduces nitric oxide synthesis in LPS-stimulated macrophages (Gannon et al., 2001). In addition, SR31747 blocked LPS-induced production of cytokines IL-1, IL-6, and TNFα in a dose-dependent manner “*in vitro*” but not “*in vivo*” (Derocq et al., 1995). SR31747 inhibits both delayed-type hypersensitivity granuloma and graft-versus-host reaction (Casellas et al., 1994). Moreover, SR31747 impairs activation of T cells and reduces IFN-γ and GM-CSF gene expression, while it barely alters IL-4 gene expression (Carayon et al., 1995). SR125329A, a σ-1/σ-2 ligand, which is also a human sterol isomerase ligand, inhibited staphylococcal enterotoxin B-induced mouse splenocyte proliferation (Bourrie et al., 2002) and enhanced LPS-induced systemic release of IL-10 while simultaneously inhibiting TNF-α synthesis. It also prevented graft-versus-host disease (Bourrie et al., 2002). Cocaine has been reported to act as σ-1 receptor ligand being able to modulate immune function *in vivo* and *in vitro* (Pellegrino and Bayer, 1998; Xu et al., 1999). Thus, cocaine modulates cytokine production by human peripheral blood lymphocytes, down-regulating the production of IFN-γ, and IL-6 in leukocytes and increasing the production of TGF-β by macrophages (Mattana et al., 1994; Mao et al., 1996, 1997) and IL-10 by splenocytes (Zhu et al., 2003), Nonetheless, some results suggest that the immunomodulatory effects of cocaine may be mediated, in part, by modification of endothelial-derived cytokine production (Mao et al., 1997). Thus, although several reports have addressed the anti-inflammatory and immunosuppressive activity of σ ligands in leukocytes, the molecular mechanisms responsible for the actions of σ ligands selective for the σ-1 or σ-2 receptor in T cells, remain unknown.

Activation of T cells triggers a complex regulatory cascade of events leading to activation of transcription factors including Nuclear Factor of Activated T Cells (NFAT) and Nuclear Factor (NF)-κB, that culminates in the induced transcription of a variety of activation-associated genes that in turn, regulate cell proliferation, differentiation, and acquisition of effectors functions by cells of the immune system (Altman et al., 1990; Ullman et al., 1990; Weiss and Littman, 1994). NFAT proteins are involved in the regulation of the transcriptional induction of a variety of genes that are essential in the immune and inflammatory response such as cytokines (IL-2, IL-4, IL-13, GM-CSF, TNFα), pro-inflammatory genes (COX-2), and cell surface receptors (FasL, CD40L; Rao et al., 1997; Crabtree, 1999; Iniguez et al., 2000).

Here, we have analyzed a panel of σ-1 and σ-2 ligands (either agonists or antagonists) in T cell function *in vitro* showing that treatment with σ-2 agonists interferes with T cell activation and cytokine gene expression. Our findings suggest that σ-2 agonists display immunomodulatory and anti-inflammatory actions through the inhibition of NFAT-dependent transcription, leading to down-regulation of T cell activation.

## Materials and Methods

### Cell culture

The Jurkat human leukemic T cell line was grown in complete RPMI 1640 medium, supplemented with 10% Fetal Bovine Serum (FBS), 2 mM Glutamine and antibiotics. Purified human peripheral blood T lymphocytes (PBTs) were obtained from partially purified human blood by Ficoll-Hypaque gradient as previously described (Iniguez et al., 1999). Cells were stimulated with Phorbol 12-myristate 13-acetate (PMA; Sigma-Aldrich; 15 ng/ml), A23187 calcium ionophore (Ion; Sigma); (1 μM). Sigma ligands: PRE-O84 2-(4-Morpholinethyl) 1-phenylcyclohexanecarboxylate (σ-1 agonist; Tocris; Su et al., 1991); BD-1063 1-[2-(3,4-Dichlorophenyl)ethyl]-4-methylpiperazine (σ-1 antagonist; Tocris; Matsumoto et al., 1995); BD-737 (1*S*,2*R*)-*cis*-*N*-[2-(3,4-dichlorophenyl)ethyl]-*N*-methyl-2-(1-pyrrolidinyl)-cyclohexylamine (Bowen et al., 1992) and CB-184 -1R, 5R-(E)-8-(3,4-dichlorobenzylidene)-5-(3-hydroxyphenyl)-2-methylmorphan-7-one (σ-2 agonists; Bowen et al., 1995) and; AC-927 N-phenethylpiperidine oxalate (σ-2 antagonist; Berardi et al., 2004), were synthesized by Laboratories Esteve. These compounds were dissolved in DMSO and tested over a range of concentrations (0.1–10 μM). Cyclosporin A (CsA, 100 ng/ml; Sigma-Aldrich) or σ ligands were added 1 h prior PMA + Ion stimulation.

### mRNA analysis

Total RNA was prepared from cells by the TRIzol reagent RNA protocol (Invitrogen). For standard RT-PCR, RNA (1 μg) was reverse transcribed by the RNA PCR core kit (Perkin-Elmer). cDNA was used for PCR amplification to analyze gene expression by standard RT-PCR using specific primers for σ-1sense (5′-CTGGATGGGCGCCATGTG-3′); σ-1-antisense (5′-GGCCAAAGAGGTAGGTGG-3′); σ-2/PGRMC1-sense (5′-ATCATGGCTGCCGAGGATGTG-3′); σ-2/PGRMC1-antisense (5′-GAGGTCAGAAAGGTCATCGTAC-3′).

GAPDHsense (5′-TGGCAAATTCCATGGCA-3′); GAPDHantisense (5′-TCTAGACGGCAGGTCAGGTCCACC-3′); IL-2sense (5′-ATGTACAGGATGCAACTCCTGTCTT-3′); IL-2antisense (5′-GTCAGTGTTGAGATGATGCTTTGAC-3′).

PCR reaction was amplified by 25–30 cycles of denaturation at 94°C for 45 s, annealing at 55 or 60°C for 45 s, and extension at 72°C for 45 s. PCR products were separated on agarose gel electrophoresis and visualized by ethidium bromide staining.

For quantitative real-time RT-PCR analysis, total RNA was reversed transcribed using the components of the High Capacity cDNA Archive Kit (Applied Biosystems). Amplification of cDNAs was performed using the TaqMan Universal PCR Master Mix (Applied Biosystems) on an ABI PRISM 7900HT instrument (Applied Biosystems) for 40 cycles with specific primers and Taqman MGB probes for IL-2, TNFα, COX-2, and GAPDH. (Applied Biosystems). All samples were run in triplicate. Quantification of gene expression by real-time RT-PCR was calculated by the comparative threshold cycle (ΔΔCT) method following the manufacturer’s instructions. Relative quantification (RQ) of mRNA levels was determined using endogenous expression of GAPDH and is shown in all the experiments as RQ ± standard deviation (SD).

### Transfection and luciferase assays

Luciferase reporter assays in Jurkat cells were performed by transient transfection of different plasmids in exponential growing cells (1 × 10^6^ cells/ml in OPTIMEM medium) by the Lipofectamine 2000 reagent (Invitrogen). After 4 h of incubation, RPMI medium containing 5% FBS was added to cells and the incubation was continued for 16 h to complete transfection. Cells were treated with increasing doses of the indicated compounds for 1 h prior to treatment with PMA (15 ng/ml) + Ion (1 μM) for additional 5 h. Luciferase activity was determined in cell lysates with a luciferase assay kit (Promega) with a luminometer Monolight 2010 (Analytical Luminescence Laboratory). Results are represented as percentage of activity, considering 100% that obtained in the PMA + Ion stimulated samples, and are shown as the mean ± SD from triplicate assays.

Human COX-2 promoter construct COX-2-Luc (p2-1900) has been described previously (Iniguez et al., 1999). The IL-2-LUC plasmid contains the region spanning from −326 to + 145 of the human IL-2 promoter (Durand et al., 1988). The plasmid TNFα-LUC contains a region 1311 bp upstream from the transcriptional initiation site of human TNFα promoter (Rhoades et al., 1992). The NFAT-LUC reporter construct contains three tandem copies of the distal NFAT binding site fused to the IL-2 minimal promoter (Durand et al., 1987). NF-κB luciferase reporter contains three tandem repeats of the NF-κB-binding site from the Igκ promoter located upstream of a minimal conalbumin promoter controlling the luciferase gene (Arenzana-Seisdedos et al., 1993).

### Cytokine measurement

The concentration of IL-2 and TNFα in supernatants of Jurkat cell cultures was determined using specific ELISAs in the presence or absence of σ ligands. Commercially available kits were used according to manufacturer’s instructions (Bender MedSystems). Cytokine concentration was assayed in triplicate.

### Prostaglandin E_2_ measurement

To evaluate PGE_2_ production in Jurkat cells after the different treatments, the media was aspirated and cells rinsed with HBS, pH 7.4, supplemented with 0.1% BSA. Then, cells were incubated for 30 min at 37°C in the same buffer with an excess of AA (10 μM). Levels of PGE_2_ were determined in the supernatants using a PGE_2_ enzyme immunoassay kit (Cayman Chemical). All samples were tested in triplicate.

### Immunoblot analysis

Total extracts of Jurkat cells were obtained after lysis for 30 min in ice-cold lysis buffer (Phosphate-buffered saline, 1% Non-idet P-40, 0.5% sodium deoxycholate, 0.1% SDS, 1 mM phenylmethylsulphonyl fluoride, 2 μg/ml aprotinin, 2 μg/ml leupeptin, and 1 μg/ml pepstatin, 1 mM sodium orthovanadate and 1 mM sodium fluoride). Lysates were denatured and resolved by SDS-polyacrylamide gel electrophoresis and then transferred to nitrocellulose membranes. The filters were incubated with anti-NFAT antisera (anti-NFATc2 #672; 1:3000; generous gift of Dr. J. M. Redondo) that recognize both the dephosphorylated and phosphorylated forms of NFAT (San-Antonio et al., 2002; Jimenez et al., 2004). β-actin levels were determined as a control of loading in each lane with a specific antibody (Santa Cruz Biotechnology). After incubation with a goat anti-rabbit IgG secondary antibody linked to horseradish peroxidase (Pierce) at 1:15000 dilution, the stained bands were visualized with the ECL (enhanced chemiluminescence) detection reagent (Pierce).

### Statistics

Results are expressed as mean ± SD from at least three independent experiments performed by duplicate or triplicate. Statistical analysis was carried out by the Student *t*-test with *p* < 0.05 representing significance. Statistical analyses were done using Graph Pad software.

## Results

### Expression of the σ receptors in human T lymphocytes

**Figure 1:**
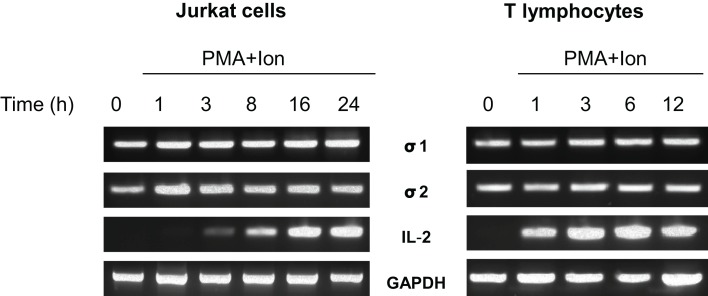
**Expression of σ receptors in human T cells**. RNA from Jurkat T cells or human T lymphocytes stimulated with PMA (15 ng/ml) + Ion (1 μM) for the indicated times (hours) was analyzed by RT-PCR to determine σ-1 and σ-2/PGRMC1 receptors, IL-2 or GAPDH mRNA levels. An aliquot of the amplified DNA was separated on an agarose gel and stained with ethidium bromide for qualitative comparison.

Expression of the σ-1 and σ-2/PGRMC1 receptors mRNAs in human T cells was analyzed by RT-PCR. As shown in Figure 1, constitutive expression of the σ-1 and σ-2/PGRMC1 receptors mRNAs were detected in Jurkat T cell line and in primary lymphocytes isolated from human blood. Expression of these receptors did not seem to be influenced by activation of the cells with the phorbol ester PMA + the Ca^++^ ionophore A23187 (Ion) at any of the times analyzed. This pharmacologic treatment is known to mimic T cell receptor activation (TCR) in T cells. IL-2 mRNA expression was induced in a time-course dependent manner, which serves as a control of gene transcriptional induction upon T lymphocyte activation. Levels of GAPDH mRNA are shown as a control of loading. These results indicate that both σ-1 and σ-2/PGRMC1 receptors are constitutively expressed in human T lymphocytes but they are not modulated by TCR activation.

### Effect of σ-1 and σ-2 ligands on cytokine production in T cells

**Figure 2:**
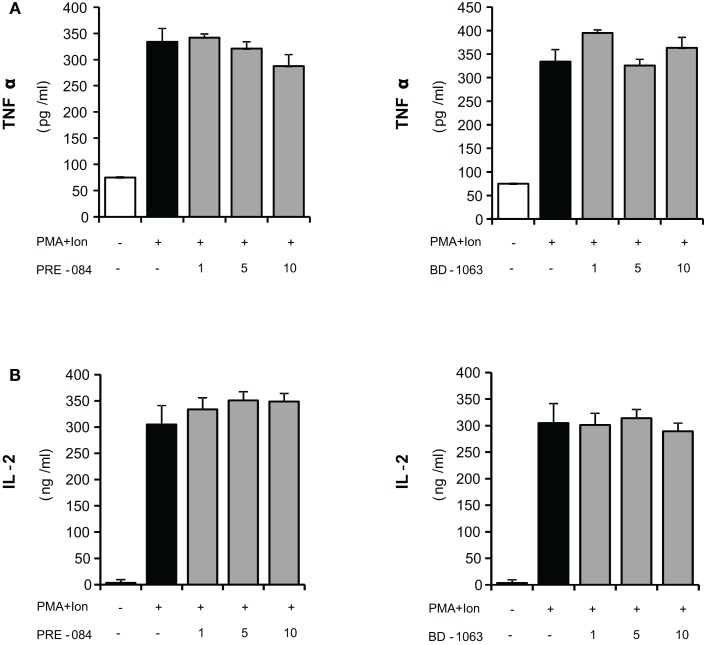
**Effects of σ-1 ligands on TNFα and IL-2 production by activated Jurkat T cells**. Jurkat cells were treated for 16 h with PMA (15 ng/ml) + Ion (1 μM) in the presence of different concentrations (1, 5, and 10 μM) of the σ-1 agonist PRE-O84 or the antagonist BD-1063. Levels of TNFα **(A)** and IL-2 **(B)** released into the medium were measured by an ELISA assay.

Activated T cells produce a variety of cytokines such as IL-2 and TNF-α, among others. The coordinated production of these cytokines is crucial for regulation of the inflammatory response. We evaluated the immunomodulatory actions of increasing doses of σ ligands on the production of IL-2 and TNFα by activated Jurkat cells. Both selective σ-1 agonist PRE-084 (*K*_i_ values of 2.2 and 13091 nM for σ-1 and σ-2 receptors respectively; Su et al., 1991; Rossi et al., 2011) and σ-1 antagonist BD-1063 (*K*_i_ value of 9 nM for the σ-1 receptor and more than 49 times selectivity over the σ-2 receptor; Matsumoto et al., 1995; Entrena et al., 2009), were used to determine the effect of σ-1 ligands in these cells. As shown in Figure 2, neither the σ-1 agonist PRE-084 nor the σ-1 antagonist BD-1063 showed a significant effect on the induction of the production of IL-2 or TNFα in Jurkat T cells upon stimulation with PMA + Ion.

**Figure 3:**
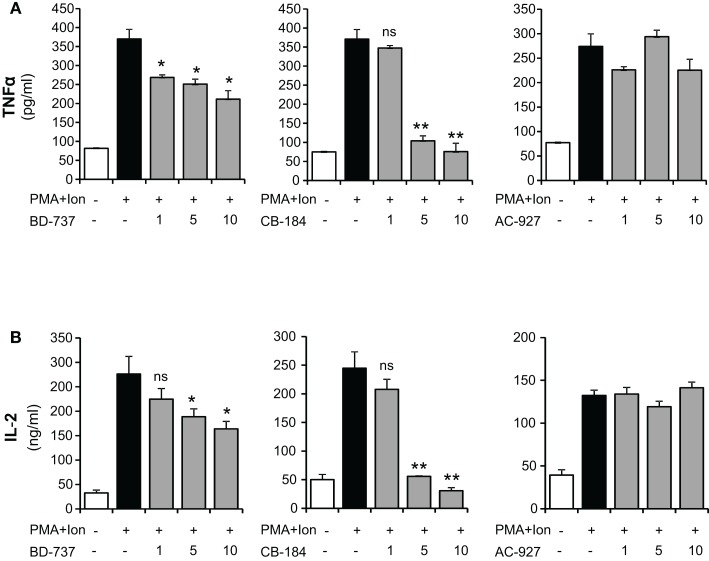
**Inhibition of TNFα and IL-2 production by σ-2 agonists in activated Jurkat T cells**. Jurkat cells were treated for 16 h with PMA (15 ng/ml) + Ion (1 μM) in the presence of different concentrations (1, 5, and 10 μM) of the σ-2 agonists BD-737 and CB-184 or the antagonist AC-927. Levels of TNFα **(A)** and IL-2 **(B)** released into the medium were measured by an ELISA assay. (n.s, not significant; *p < 0.05; **p < 0.01 versus PMA + Ion).

We next evaluated the effects of different σ-2 ligands in these cells: CB-184, described as a selective σ-2 receptor agonist with 554-fold selectivity for σ-2 receptors over σ-1 (Bowen et al., 1995; Vilner and Bowen, 2000; Crawford et al., 2002); BD-737, a potent σ receptor ligand with properties as σ-2 ligand (Bowen et al., 1992; Vilner and Bowen, 2000; Crawford et al., 2002); and the σ-2 receptor antagonist AC927 (Crawford et al., 2002; Berardi et al., 2004; Matsumoto et al., 2008). Different to σ-1 ligand, both σ-2 agonists, BD-737, and CB-184, produced a dose-dependent decrease in the PMA + Ion-mediated induction of IL-2 or TNFα production, with the strongest effect observed after treatment with CB-184. The σ-2 antagonist AC-927 did not show a significant effect at any of the doses assayed (Figure 3).

**Figure 4:**
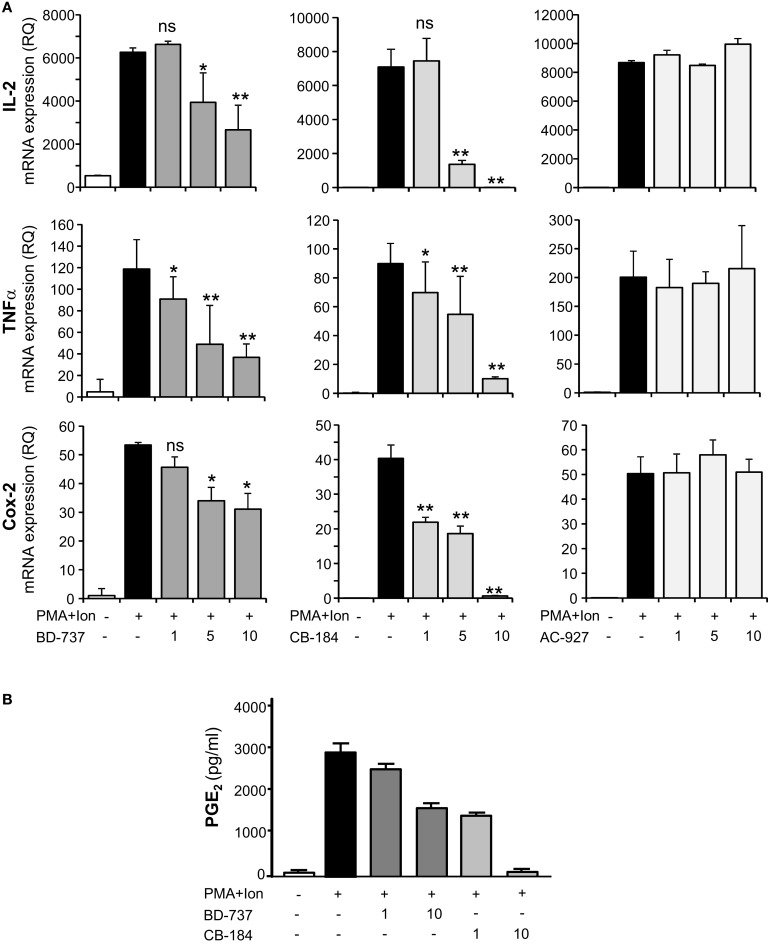
**Effect of σ-2 ligands on the expression of IL-2, TNFα, and COX-2 in Jurkat T cells**. **(A)** Total RNA from Jurkat cells, treated with σ-2 agonists BD-737 and CB-184 or the antagonist AC-927 at the doses indicated (1, 5, and 10 μM) and grown in the absence or presence of PMA (15 ng/ml) + Ion (1 μM), was analyzed by quantitative RT-PCR. Relative quantification (RQ) of mRNA levels of IL-2, TNFα, and COX-2 was determined using endogenous expression of GAPDH and is shown as RQ ± SD. **(B)** PGE_2_ production by Jurkat cells treated with σ-2 agonists BD-737 and CB-184 at the doses indicated (1 and 10 μM), grown in the absence or presence of PMA + Ion. PGE_2_ was measured by a standard EIA assay as described in Section “*Materials and Methods*.” Results are shown as the mean ± SD of PGE_2_ in pg/ml of determinations conducted in triplicate. (n.s, not significant; *p < 0.05; **p < 0.01 versus PMA + Ion).

To determine whether the effects of sigma ligands on cytokine production were a consequence of changes in mRNA expression, we performed quantitative real-time RT-PCR assays, analyzing mRNA levels of these cytokines as well as of another gene involved in inflammation as cyclooxygenase (COX)-2. In agreement to the above results, increasing doses of σ-2 agonists significantly diminished the induction by PMA + Ion of TNFα, IL-2, or COX-2 mRNAs (Figure 4A). No effect of the σ-2 antagonist AC-927 was observed. The inhibitory effects of these σ-2 agonists on COX-2 expression correlated with those observed with COX activity. Thus, BD-737 and CB-184 produced a dose-dependent decrease in the PMA + Ion-mediated induction of PGE_2_ production in Jurkat cells (Figure 4B).

**Figure 5:**
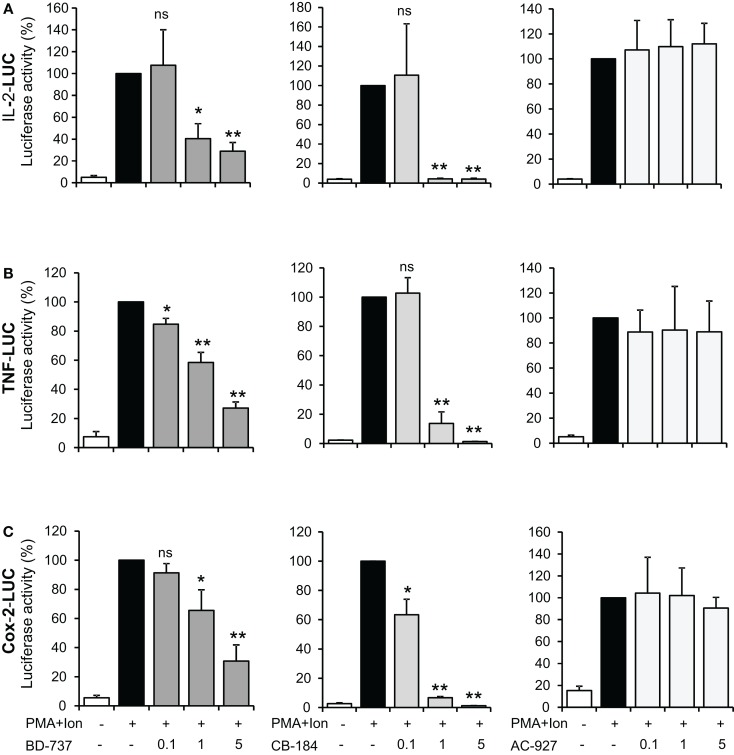
**Inhibitory effect of σ-2 agonists on the activity of IL-2, TNFα, and COX-2 promoters in Jurkat T cells**. Jurkat cells were transiently transfected with luciferase reporter constructs containing the promoter regions of the IL-2 **(A)**, TNFα **(B)**, or COX-2 **(C)** genes and treated for 6 h with PMA (15 ng/ml) + Ion (1 μM) in the absence or presence of increasing doses (0.1, 1, and 5 μM) of σ-2 ligands BD-737 and CB-184 (σ-2 agonists) or AC-927 (σ-2 antagonist) as indicated. Luciferase activity was measured and represented in percentage of activity considering 100% the activity obtained upon PMA + Ion stimulation in the absence of σ ligand. (n.s, not significant; *p < 0.05; **p < 0.01 versus PMA + Ion).

**Figure 6:**
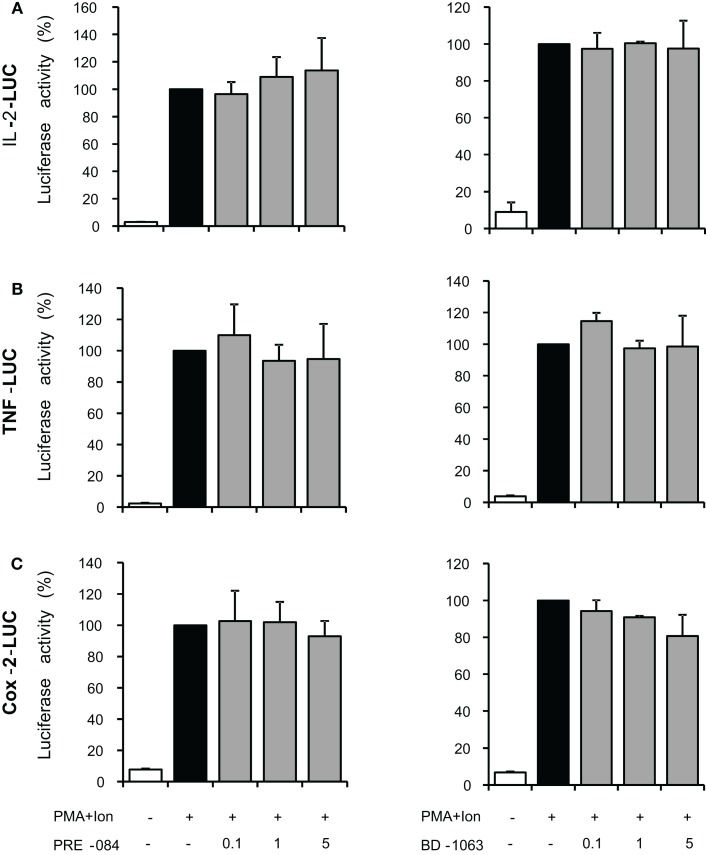
**Effect of σ-1 ligands on the activity of IL-2, TNFα, and COX-2 promoters in Jurkat T cells**. Jurkat cells were transiently transfected with luciferase reporter constructs containing the promoter regions of the IL-2 **(A)**, TNFα **(B)**, or COX-2 **(C)** genes and treated for 6 h with PMA (15 ng/ml) + Ion (1 μM) in the absence or presence of increasing doses (0.1, 1, and 5 μM) of the σ-1 agonist PRE-084 or σ-1 antagonist BD-1063 as indicated. Luciferase activity was measured and represented in percentage of activity considering 100% the activity obtained upon PMA + Ion stimulation in the absence of σ ligand.

Changes in mRNA levels can take place by the influence of these ligands on transcriptional induction, so we next investigated the regulation of TNFα, IL-2, and COX-2 promoters in Jurkat cells. As shown in Figure 5, treatment with PMA + Ion induced a substantial increase in the transcriptional induction mediated by these promoters in Jurkat T cells. Treatment with σ-2 agonists, BD-737, and CB-184, but not with the σ-2 antagonist AC-927, was able to severely impair transcriptional activation mediated by PMA + Ion in Jurkat T cells. Again, no changes in the activity of these promoters upon PMA + Ion were observed by treatment with σ-1 ligands (Figure 6).

### Effects of σ-1 and σ-2 ligands on NF-κB and NFAT-dependent transcriptional activation in T cells

**Figure 7:**
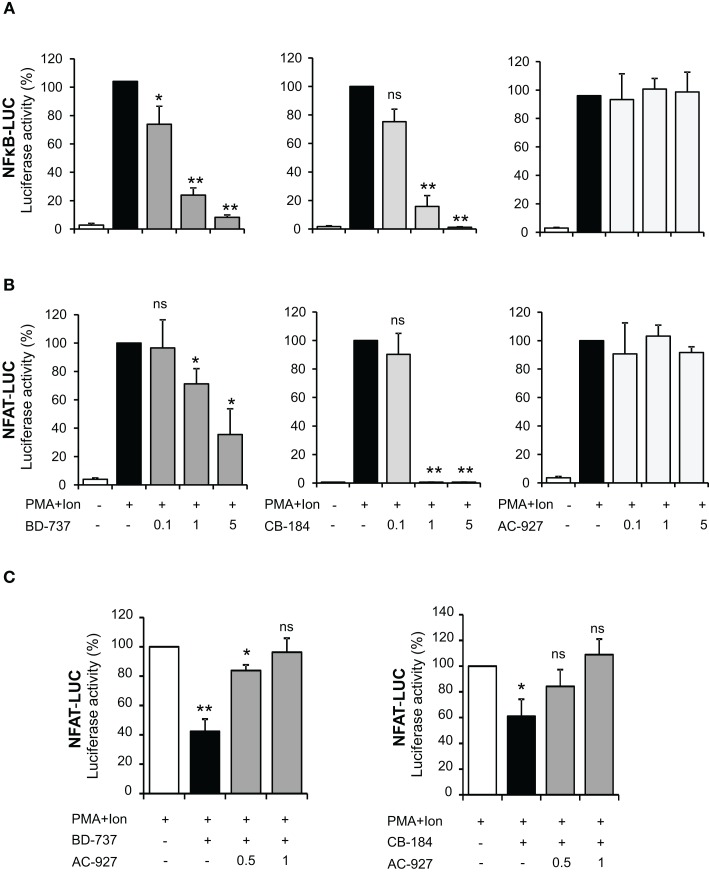
**Effect of σ-2 ligands on transcriptional induction mediated by NF-κB and NFAT in Jurkat T cells**. Jurkat cells were transiently transfected with the luciferase reporter constructs NF-κB-LUC **(A)** or NFAT-LUC **(B)** and treated for 6 h with PMA (15 ng/ml) + Ion (1 μM) in the absence or presence of increasing doses (0.1, 1, and 5 μM) of σ-2 agonists (BD-737 and CB-184) or antagonist (AC-927). **(C)** Cells transfected with the NFAT-LUC plasmid and treated with PMA + Ion were treated with σ-2 agonists BD-737 (5 μM) or CB-184 (0.5 μM) in the absence or presence of increasing doses of σ-2 antagonist AC-927 (0.5 and 1 μM). Luciferase activity was measured and represented in percentage of activity considering 100% the activity obtained upon PMA + Ion stimulation in the absence of σ ligands. (n.s, not significant; *p < 0.05; **p < 0.01 versus PMA + Ion).

**Figure 8:**
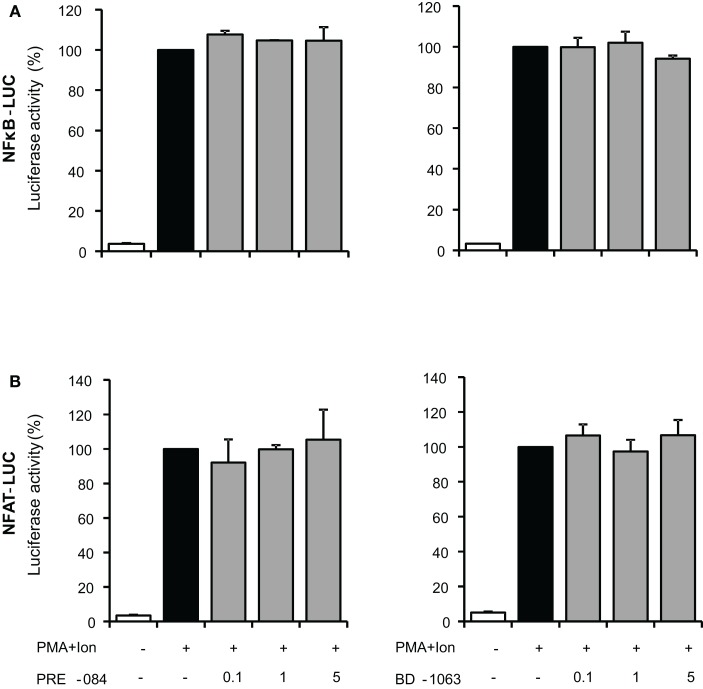
**Effect of σ-1 ligands on transcriptional induction mediated by NF-κB and NFAT in Jurkat T cells**. Jurkat cells were transiently transfected with the luciferase reporter constructs NF-κB-LUC **(A)** or NFAT-LUC **(B)** and treated for 6 h with PMA (15 ng/ml) + Ion (1 μM) in the absence or presence of increasing doses (0.1, 1, and 5 μM) of the σ-1 agonist (PRE-084 and CB-184) or antagonist (AC-927). Luciferase activity was measured and represented in percentage of activity considering 100% the activity obtained upon PMA + Ion stimulation in the absence of σ ligands.

Transcriptional activation of a variety of genes induced upon T cell activation, including TNF-α, IL-2, and COX-2, depends on the coordinate interactions among several transcription factors, including members of NF-κB, and NFAT families (Rao et al., 1997; Crabtree, 1999). To determine whether inhibition of gene expression was occurring through the action of σ ligands on the activation of these transcription factors, we evaluated the effect of these compounds on the activity of NF-κB and NFAT by using luciferase reporter gene constructs including minimal promoters under the control of NFAT or NF-κB-binding sites. Treatment with PMA + Ion promoted an important increase in the activity of both NF-κB-LUC and NFAT-LUC reporter constructs in Jurkat T cells. Figures 7A,B shows the results obtained with the σ-2 agonists BD-737 and CB-184 and σ-2 antagonist AC-927. Sigma-2 agonists treatment displayed a profound inhibitory effect on transcriptional activation mediated by both NF-κB and NFAT. In contrast, σ-2 antagonist AC927 or σ-1 ligands PRE-084 and BD-106 did not exhibit any significant effect on the activity of these transcription factors (Figures 7 and 8).

**Figure 9:**
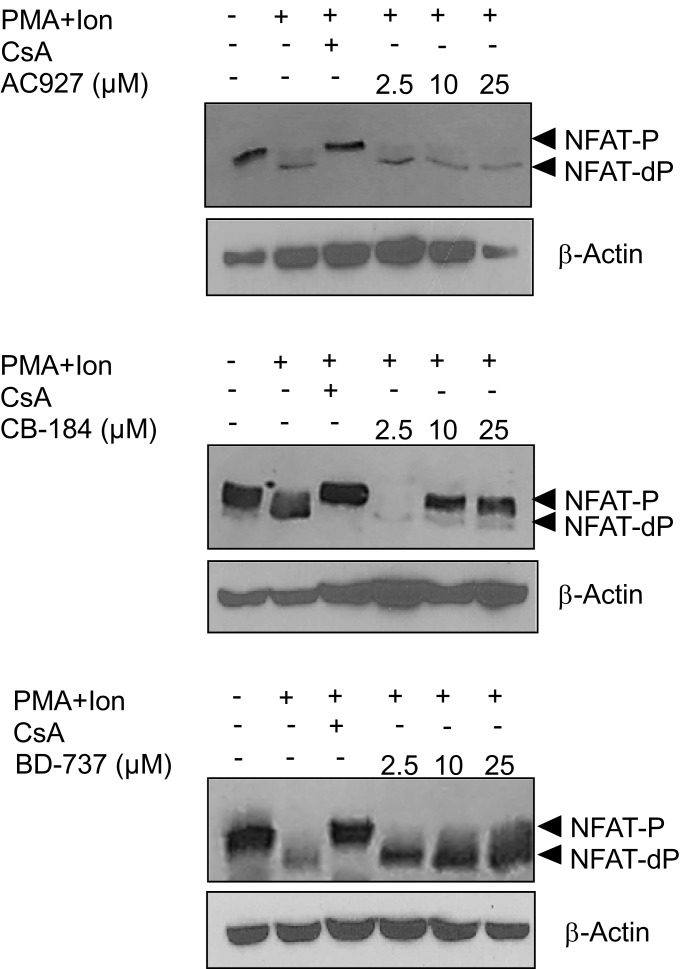
**Actions of σ 2 ligands on NFAT dephosphorylation**. Protein extracts from Jurkat treated with PMA (15 ng/ml) + Ion (1 μM) for 2 h in the presence or absence of increasing doses (2.5, 10, and 25 μM) of σ-2 agonists (BD-737 and CB-184) or antagonist (AC-927) were analyzed by Western blot with an anti-NFAT serum. The phosphorylated (NFAT-P) and dephosphorylated (NFAT-dP) forms of NFATc2 are indicated by arrows. Inhibition of NFAT dephosphorylation by Cyclosporin A (CsA) is shown as a control. β-Actin protein levels were determined as a control of loading.

Inhibitors of NFAT activation as Cyclosporine A or Tacrolimus are well-known immunomodulatory drugs with a potent immunosuppressive effect (Kiani et al., 2000; Martinez-Martinez and Redondo, 2004). The above results pointed to σ-2 agonists as able to interfere with NFAT-dependent transcriptional activation of gene expression. To further confirm the specificity of the actions of σ-2 agonists on NFAT activation, we tested whether a σ-2 antagonist could block this effect. As shown in Figure 7C, the inhibitory effect of both BD-737 and CB-184 on NFAT-LUC was reversed in a dose-response manner by the σ-2 antagonist AC-927. To address the mechanism involved in the actions of these compounds on NFAT-dependent transcription, we next analyzed the effect of these compounds on NFAT phosphorylation. In resting T cells, NFAT is a cytoplasmic factor that upon activation is dephosphorylated by the calcineurin phosphatase and translocated into the nucleus leading to transactivation of target genes (Rao et al., 1997; Crabtree, 1999; Hogan et al., 2003). As shown in Figure 9, upon activation with PMA + Ion NFAT is dephosphorylated, showing a decrease in phosphorylated NFAT signal and an increase in a band of dephosphorylated NFAT, which migrates faster in SDS-PAGE. Both BD-737 and CB-184 partially inhibited in a dose-dependent manner the dephosphorylation of NFAT. In contrast, AC 927 had no effect. As a control it is shown the inhibition of calcineurin phosphatase activity by Cyclosporin A (CsA) and hence, NFAT dephosphorylation, which is known to result in a complete prevention of nuclear translocation of NFAT (Kiani et al., 2000; Martinez-Martinez and Redondo, 2004). This result pointed to σ-2 agonists as inhibitor of NFAT signaling, interfering with the early steps in the activation of this factor.

Taken together, our results support an inhibitory role of NFAT-mediated transcriptional activation in Jurkat T cells by the σ-2 agonists BD-7373 and CB-184, which, in addition to inhibition of NF-κB activation, could account for the inhibitory actions on the transcriptional induction and expression of activation-induced genes such as TNFα, IL-2, and COX-2.

## Discussion

In this report we have analyzed the effect of σ ligands on T cell function. Our results show a novel pharmacological activity of sigma-2 ligands on the immune system. Namely, σ-2 agonists have immunosuppressive activity in T lymphocytes. On the other hand, signaling through the σ-1 receptor, at least by the results obtained with the selected σ-1 agonists or antagonists, does not seem to exert evident functional consequences on the immune activation parameters assayed. We found that basal levels of σ-1 receptor mRNA are present in resting human T cells that do not change upon T cell activation. A similar profile of expression was observed with the recently described putative σ-2 receptor PGRMC1 (Xu et al., 2011). Expression of this receptor has been described in peripheral blood cells and T lymphocytes, being elevated in multiple tumor types (Mir et al., 2012; Ndiaye et al., 2012). The existence of a σ binding site in the Jurkat human T cell line corresponding to an alternatively spliced σ-1 receptor variant has been previously described (Ganapathy et al., 1999). Nonetheless, pharmacology of the sigma binding site in Jurkat T cells seems to be consistent with the σ-2 receptor subtype (DeHaven-Hudkins et al., 1996).

Several reports have described anti-inflammatory and immunoregulatory properties of σ ligands both “*in vitro*” and “*in vivo*” (Casellas et al., 1994; Bourrie et al., 1995, 2002; Carayon et al., 1995; Derocq et al., 1995; Liu et al., 1995; Gannon et al., 2001; Zhu et al., 2003). However the exact biological significance and the nature of σ receptor signaling mediating these effects are poorly understood. Several of the anti-inflammatory and immunosuppressive effects of sigma ligands in immune cells have been suggested to be indirect, due to their ability of these compounds to induce anti-inflammatory cytokines as TGFβ or IL-10 (Bourrie et al., 1995; Zhu et al., 2003; Kvaratskhelia et al., 2009) or even by promoting glucocorticoid-dependent immunosuppression (Derocq et al., 1995). Moreover, controversial data about the identification of these effects as selectively dependent on σ-1 or σ-2 exist. There is a lack of consistency between pharmacology of σ binding selectivity and the immunomodulatory events observed in response to σ ligands. These discrepancies may be due to the different σ compounds used or to the fine specificity of those ligands for different unidentified target receptors in immune cells. Kvaratskhelia et al. (2009) have reported that σ-ligands can modulate IL-8 and IL-10 production by T cells through modulation of the NMDA Glutamate Receptor. Particularly, very little is known about the pharmacology of σ-2 receptors and the effect of ligands acting specifically through this receptor. The fact that the real nature of the σ-2 receptor has not been clearly determined has prevented further analysis. Signaling cascades directly coupled to activation of this receptor have not yet been identified and only some data is available on its function.

Here we provide some new data on σ-2 receptor function and signaling. Thus, we have found that the σ-2 receptor agonists BD-737 and CB-184 had an interesting behavior compatible with being immunosuppressive and anti-inflammatory drugs. Since our results show that two σ-2 receptor agonists with different chemicals properties have a similar effect which is reversed by a σ-2 antagonist, it is likely that a putative σ-2 receptor binding site participates in the observed effect in T lymphocytes. In contrast to σ-1 compounds, here, we have shown that σ-2 agonists but not just a merely σ-2 ligand as the σ-2 antagonist AC927 are able to decrease T cell activation “*in vitro*”. Those two σ-2 agonist compounds of different chemical structure inhibited COX-2, TNFα, and IL-2 mRNA as well as cytokine release by activated T cells. Those effects seem to occur through inhibition of transcriptional activation of these genes as σ-2 agonists were able to diminish the PMA + Ion-mediated induction of the activity of TNFα, IL-2, and COX-2 promoters in T cells. This effect takes place at the transcriptional level, reducing NFAT and NF-κB activation. We present evidence showing that BD-737 and CB-184 inhibited PMA + Ion-mediated activation of NFAT, altering its dephosphorylation and hence nuclear shuttling and NFAT-mediated gene transcription. Interestingly, the effects on NFAT-dependent transcription can be prevented by a σ-2 antagonist, further confirming the specificity of action. NFAT proteins play an important role in many cellular processes being involved in the regulation of the expression of a variety of T cell cytokines (IL-2, IL-4, IL-5, IL-6, IL-8, IL-13, IFN-γ, TNF-α) essential for the immune response and inflammation (Rao et al., 1997; Crabtree, 1999; Hogan et al., 2003). Thus, inhibition of NFAT activation by σ-2 agonist may explain their effects on transcriptional regulation of NFAT-dependent genes. Furthermore, our results show that the σ-2 agonists evaluated inhibited NFAT dephosphorylation, an essential step for NFAT activation and entry into the nucleus (Rao et al., 1997; Crabtree, 1999; Hogan et al., 2003). Treatment with increasing doses of σ-2 agonist causes NFAT to be maintained in a phosphorylated state and hence block the nuclear translocation of NFAT and, thus NFAT-dependent gene transcription. Whether this effect is occurring by a mechanism involving interference on Ca^++^ signaling or calcineurin phosphatase activity as in the case of the well-known NFAT inhibitors CsA and Tacrolimus (Kiani et al., 2000; Martinez-Martinez and Redondo, 2004), remains to be elucidated. Besides, NFAT activation plays a promoting role in cancer (Duque et al., 2005; Viola et al., 2005; Gachet and Ghysdael, 2009). Thus, it is tempting to speculate that some of the effects of σ-2 agonists on cancer may take place though NFAT inhibition. Further studies are needed to elucidate this hypothesis.

In summary, our results show that σ-2 agonist-ligands have an important immunosuppresor and anti-inflammatory effect inhibiting T cell activation at relevant pharmacological doses by blocking transcriptional activation through their interference with NF-κB and especially NFAT activation. Nonetheless, our data will not necessarily predict the effects of σ-2 agonists *in vivo*, further. Therefore, these data open a new field for the study of σ-2 ligands as new therapeutic agents in pathologies as inflammatory and autoimmune diseases. In addition, they also open new answers to explore the therapeutic effect of σ-2 agonists on other diseases where NFAT inhibition may play a pathological role.

## Conflict of Interest Statement

Javier Burgueño and José M. Vela are employes of Laboratories ESTEVE, which partially funded this research.

## Abbreviations

COX, Cyclooxygenase; CsA, Cyclosporin A; Ion, A23187 calcium ionophore; FBS, fetal bovine serum; GAPDH, glyceraldehyde 3 phosphate dehydrogenase; IL, interleukin; Luc, luciferase; NFAT, nuclear factor of activated T cells; NFκB, nuclear factor κB; PBTs, peripheral blood T lymphocytes; PG, prostaglandin; PMA, phorbol 12-myristate 13-acetate; SD, standard deviation; TCR, T cell receptor; TNF tumor necrosis factor.
